# Avian Leukosis Virus Subgroup J in Layer Chickens, China

**DOI:** 10.3201/eid1610.100780

**Published:** 2010-10

**Authors:** Yu-Long Gao, Li-Ting Qin, Wei Pan, Yong-Qiang Wang, Xiao-Le Qi, Hong-Lei Gao, Xiao-Mei Wang

**Affiliations:** Author affiliation: Harbin Veterinary Research Institute–Chinese Academy of Agricultural Sciences, Harbin, People’s Republic of China

**Keywords:** Avian leukosis virus, subgroup J, layer chicken, epidemiology, China, viruses, letter

**To the Editor:** In recent years, cases of avian leukosis virus subgroup J (ALV-J) infection and tumors in commercial layer chickens and breeders of egg-type chickens have been emerging in the People’s Republic of China. ALV-J was first isolated from meat-type chickens with myeloid leukosis in 1988. Although egg-type chickens have been experimentally infected with ALV-J to induce tumors (*1*), field cases of ALV-J infection and tumors in commercial layer chickens were not found worldwide until 2004 (*2*).

ALV-J has recently been found to have induced various tumors and caused production problems in commercial layer flocks and local chicken breeds in China (*2**,**3*). Many field cases of ALV-J infection and tumors have occurred in 15- to 29-week-old egg-type chickens in several provinces. Affected flocks had dramatically reduced egg production and hemorrhage in the skin surrounding the phalanges and feather follicles. Some birds had gray-white nodules in the liver, spleen, or kidneys, and liver and spleen were enlarged up to several times their normal size. Morbidity rates for some flocks reached 60%, and mortality rates for some flocks were >20%. Clinical samples from livers, spleens, whole blood, and tumors were collected from chickens in different provinces and sent for laboratory diagnosis. Results showed that the predominant virus in the samples was ALV-J.

During 2007–2009, we conducted an epidemiologic investigation of ALV in layer flocks in China. All virus isolation was performed in DF-1 cells. Briefly, 233 clinical samples were collected from 44 layer flocks in different provinces and used to inoculate subconfluent cell cultures containing Dulbecco modified essential medium supplemented with 10% (vol/vol) fetal bovine serum and antimicrobial drugs. After a 7–9 day incubation period, the cells were frozen and thawed 3×. A group-specific antigen-capture ELISA was used to identify ALV. After proviral DNA was extracted directly from infected cell culture or tumors, PCR with strain-specific primers was used to detect ALV-A, ALV-B, or ALV-J (*4*).

Of these samples, 150 (64.4%) were ALV-J positive, 28 (12.1%) were ALV-A positive, and 8 (3.4%) were ALV-B positive. Phylogenetic analysis showed an 87.3%–98.2% aa sequence identity of *env* genes in all ALV isolates compared with the HPRS-103 strain (*5*). All isolates had complete repeated transmembrane deletion and partial direct repeat–1 deletion but contained an intact E element. A mutation was found in the enhancer and promoter region of the U3 region in the 3′ long terminal repeat; this mutation is not found in ALV-J isolated from broiler chickens (*6*).

The newly isolated ALV-J strain from layer chickens was used to examine the pathogenicity in 1-day-old White Leghorn specific pathogen–free chicks soon after hatching in separate incubators and rooms in the experimental animal house facilities at Harbin Veterinary Research Institute, Harbin, China. The chicks were inoculated intraabdominally with a 1,000-unit 50% tissue-culture infective dose of ALV-J propagated in the DF-1 cells. Blood samples were collected to check for viremia at 10 weeks of age. Experimental birds were reared until 27–30 weeks of age.

Prolonged viremia developed in 15 (50%) of 30 chicks; hemangiomas developed in the skin surrounding phalanges and in the liver of 3 (10%); and myeloid leukosis, detected by gross or histologic examination, developed in 10 (30.3%). A previous study showed that meat-type birds infected with ALV-J retained a high level of viremia over their lifetime (*7*) but that layer chickens cleared the infection within a few weeks. Our study demonstrated that ALV-J infection can cause disease in layer chickens and can induce tumors and long-lasting viremia. For this reason, disease caused by ALV-J in layer chickens in China should be further investigated.

Because ALV-J is vertically transmitted from dam to progeny by the embryo, it represents a potential threat for humans who receive vaccines that are produced in chicken embryonic fibroblasts or embryonated eggs (e.g., yellow fever vaccine and measles and mumps vaccine) (*8*). An effective vaccine against ALV is not available. Eradication of ALV-J has been difficult because of substantial genetic and antigenic variation among ALV-J isolates as well as high levels of vertical and horizontal transmission (*9**,**10*). Therefore, effective prevention and elimination measures should be developed as soon as possible.
